# Editorial on Special Issue “Lipid Nanosystems for Local Drug Delivery”

**DOI:** 10.3390/pharmaceutics15071970

**Published:** 2023-07-18

**Authors:** José Catita, Carla M. Lopes

**Affiliations:** 1 Instituto de Investigação, Inovação e Desenvolvimento (FP-I3ID), Biomedical and Health Sciences Research Unit (FP-BHS), Faculdade Ciências da Saúde, Universidade Fernando Pessoa, 4200-150 Porto, Portugal; 2 Paralab, SA, 4420-392 Valbom, Portugal; 3 Associate Laboratory i4HB—Institute for Health and Bioeconomy, Faculty of Pharmacy, University of Porto, 4050-313 Porto, Portugal; 4 UCIBIO—Applied Molecular Biosciences Unit, MEDTECH, Laboratory of Pharmaceutical Technology, Department of Drug Sciences, Faculty of Pharmacy, University of Porto, 4050-313 Porto, Portugal

Nanosystems provide an attractive approach to pharmacological therapy, with the possibility of enhancing the performance and overcoming the constraints of conventional therapies, thus adding substantial value to some of the already available formulations. Among these, lipid-based nanosystems (e.g., liposomes, solid lipid nanoparticles, nanostructure lipid carriers, and nanoemulsions) possess enormous potential due to their ability to improve drug delivery, enhance therapeutic efficacy, and enable the targeted and controlled release of bioactive compounds. Their unique properties make them suitable for an extensive range of applications owing to their lipid biocompatibility, low toxicity, and versatility. Currently, the development of nanomedicines that are based on lipid nanosystems capable of delivering therapeutic agents to a desired site in the body is an attractive area of pharmaceutical research. The major advantage of local drug delivery is that drug/bioactive concentrations can be enhanced in a specific desired site, minimizing toxicity to other nontargeted locations. Local drug delivery using nanosystems can be accomplished via various means: direct local placement (e.g., local administration at the intended site of use) or systemic administration, which can be either targeted or triggered.

This Special Issue of *Pharmaceutics*, entitled *Lipid Nanosystems for Local Drug Delivery*, presents a collection of ten innovative works—seven original research studies and three review papers—describing recent advances in the development of lipid nanosystems suitable for local drug delivery, employing various strategies (i.e., localized delivery, targeted/triggered strategies), and highlighting their advantages in this research area.

Concerning localized drug delivery lipid-based nanosystems, Smoleński et al. [1], in a collaboration between the Faculty of Pharmacy of Wroclaw Medical University in Poland and the University of Lille, Inserm, in France, demonstrated the potential technological properties of clotrimazole-loaded nanoemulsions for vaginal drug delivery in order to manage local vaginal disorders such as candidiasis. In this study, stable nanoemulsions with appropriate attributes in terms of their mean droplet size, homogeneity, pH, and osmolality were developed, and a pilot study was also performed in order to evaluate their release kinetics, taking into consideration the limited solubility of clotrimazole.

Although the topical administration of therapeutic agents to the skin appears to be preferable when aiming to manage skin conditions due to the lower likelihood of systemic adverse effects, the stratum corneum restricts their penetration. Therefore, lipid-based nanosystems have been demonstrated as one of the most effective strategies for improving skin permeability by fluidizing the lipid matrix of the stratum corneum without interfering with the skin barrier’s functions. In this regard, a team of researchers from the University of Huddersfield, UK, formulated a stable nanoemulsion for the topical delivery of mupirocin, a topical antibacterial agent, to the skin [2]. The authors revealed that the developed nanoemulsions had a significantly greater mupirocin permeability than the control, a commercial cream. They also reported that the nanoemulsions developed deposed mupirocin across various timespans and retained the agent in the skin depending on the essential oil used in their preparation. The study’s findings revealed the possibility of obtaining a nanoemulsion for the acute or prophylactic management of topical infections in the skin by selecting the most appropriate lipid-phase composition.

In another study involving Romanian researchers, Scomoroscenco et al. [3] prepared a novel gel microemulsion system with enhanced penetration properties (i.e., which provided a gradual and controlled release, ensuring the prolonged effect of the treatment) that is intended for the topical delivery of curcumin to the skin, employing skin-safe and biodegradable ingredients, and a low concentration of nontoxic surfactants (Plantacare combined with Akypo).

Researchers from different institutes in Jordan (Al-Zaytoonah University of Jordan, Zarqa University, Jordan University of Science and Technology, Al-Ahliyya Amman University) proposed a combined system of prednisolone-loaded microemulsion and thermoresponsive in situ microgels in order to achieve sustained local drug delivery to the eye [4]. The developed delivery system exhibited adequate mechanical, mucoadhesive, and release properties, therefore prolonging the ocular retention time and enhancing the stability of the microgel. Additionally, prednisolone microgels revealed their tolerability since they did not irritate the mucosal tissues, highlighting their potential as a vehicle for the local ocular delivery of prednisolone.

In terms of cancer therapy, several researchers are focusing on therapeutic delivery systems that regulate chemotherapeutic release and uptake, specifically in the tumor site, while limiting toxicity in healthy cells. With this perspective, a collaboration of Portuguese researchers developed and optimized pH-triggered hybrid (polymeric/lipid) lyotropic non-lamellar liquid crystalline nanoassemblies for drug delivery that exhibited the sustained and significantly higher release of doxorubicin in a pH of 5.5—i.e., an acidic environment typical of cancer cells and endosomal vesicles—compared to the release of doxorubicin under a pH of 7.5—i.e., typical of normal physiological conditions [5]. Additionally, doxorubicin-loaded hybrid nanoassemblies increased cytotoxicity in comparison to the doxorubicin-free nanoassemblies in three different tumor cell lines.

Furthermore, in a collaboration between the Humboldt University and the Potsdam Institute for Climate Impact Research, Germany, the Saratov State University and the Russian Academy of Sciences, Russia, and Aston University, UK, Semyachkina-Glushkovskaya et al. [6] proved that liposomes may be delivered intranasally into mouse brain tissues and reach glioblastoma, providing a non-invasive approach utilizing lipid-based nanosystems and near-infrared laser-based methods (photostimulation). The authors revealed the significance of the meningeal lymphatic network in the intranasal delivery of liposomes to the brain. The results revealed that the intranasal transport of liposomes to the brain reduced glioblastoma, which was considerably improved by the photostimulation of lymphatic arteries in the cribriform plate and meninges.

As is well known, different variables, such as the resistance mechanisms and physicochemical properties of antiviral agents, have a negative impact on the efficacy of antiviral therapeutic strategies. Therefore, researchers from Rhodes University in South Africa and from the University of Kinshasa and the Official University of Bukavu in the Republic of the Congo produced stable lipid nanocapsules in order to enhance the biopharmaceutical properties of efavirenz, a non-nucleoside reverse-transcriptase inhibitor [7]. The authors demonstrated effective drug encapsulation, as well as the prolonged release of efavirenz from the lipid matrix.

This Special Issue’s review papers have addressed significant themes for researchers working in the following areas of interest: (i) the application of microfluid technology to prepare lipid-based nanosystems [8]; (ii) the application of lipid nanosystems intended for topical and transdermal drug delivery [9]; and (iii) the utilization of antibody-conjugated lipid nanosystems in anticancer drug delivery to achieve an active targeting strategy.

Voogelar et al. [8], from the University of Southern California (USA), explored the utilization of microfluidics as an alternative method for manufacturing different types of lipid-based nanosystems. Microfluidics may redress several limitations of the conventional manufacturing methods, such as developing reproducible lipid nanosystems, manipulating the size of nanosystems with simple microfluidic parameter adjustments, their low cost, and high yields. Another review discusses the principal parameters that determine the permeation of nanoparticles through the skin and the applications of lipid nanosystems for cutaneous and transdermal distribution, as well as regulatory aspects [9]. In this review, Akombaetwa et al. (in a collaborating research team from Zambia, the Democratic Republic of the Congo, Belgium, and South Africa) also presented a perspective on ways in which to enhance treatment outcomes using multiple technologies, including loading the lipid nanosystems in nanofibers, hydrogels, or microneedles. Active targeting drug delivery is another crucial theme that has been approached in the cancer treatment context. In relation to this, Marques et al. [10] (from the University of Porto, Portugal) presented an insight into anticancer drug delivery employing antibody-functionalized lipid-based nanoparticles, emphasizing the application of antibody targeting ligands and the most prevalent conjugation approaches. This review also presents an update on the potential candidates that have progressed to clinical trials, as well as the possible causes for limited translation success.

The editors of this Special Issue are very grateful and express their appreciation to all of the authors, who are experts in the field of lipid-based nanosystems and have responded to our invitation to share the findings of their excellent quality research, as well as for the critical assessments of their manuscripts.
